# Portal vein thrombosis after aortic valve replacement surgery in a patient with antithrombin III deficiency - case presentation

**DOI:** 10.1186/1749-8090-9-73

**Published:** 2014-04-28

**Authors:** Yu-qing Wang, Qiu-lin Chen, Da Zhu, Li Dong

**Affiliations:** 1 Department of Cardiac Surgery, West China Hospital, Sichuan University, No. 37, Guoxue Alley, Chengdu, Sichuan Province 610041, China

**Keywords:** Portal vein thrombosis, Aortic valve replacement, Antithrombin III

## Abstract

We presented an unique case of portal vein thrombosis (PVT) after aortic valve replacement due to antithrombin III (ATIII) deficiency. PVT after aortic valve replacement (AVR) is a serious complication, which has not previously been reported.

## Background

Portal vein thrombosis (PVT) is a rare but well-known complication after abdominal surgery. Myeloproliferative disorder, liver cirrhosis with portal hypertension, deficiency of natural anticoagulant proteins such as protein C or ATIII as well as hepatocellular carcinoma are the most frequent causes of portal vein thrombosis [1]. Cardiopulmonary bypass (CPB) has long been recognized as one of the major causes of the systemic inflammatory response, which may contribute to post-operative complications such as multiple organ failure [2] as well as coagulation or hemostasis dysfunction. However, evidence about portal vein thrombosis after open heart surgery is rare and clinical experience in diagnosis and PVT management after cardiac surgery are still limited. In this article, we then presented an unique case of PVT after aortic valve replacement due to antithrombin III deficiency.

## Case presentation

A 45-year-old Chinese man presented to our hospital due to 1 year history of dyspnea on exertion, angina. Physical exam revealed an systolic murmur in left second intercostal space as well as mild degree hepatomegaly. EKG showed marked left ventricular hypertrophy. Echocardiogram confirmed severe degree aortic stenosis with congenital bicuspid aortic valve. Complete blood count showed mild-moderate degree thrombocytopenia. Routine coagulation screening test (including PT, APTT as well as fibrinogen) was also unremarkable with normal plate function and further bone marrow biopsy examination revealed no obvious abnormalities. His liver function was also normal and further abdominal ultrasound also precluded the pathology of the live.

**Figure 1:**
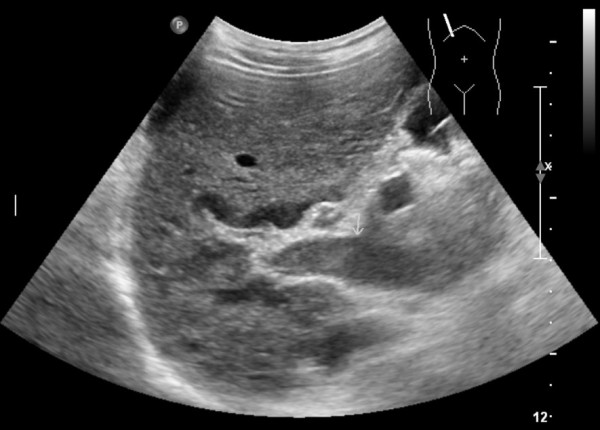
Abdominal ultrasound revealed thrombosis in proximal portion of the portal vein.

**Figure 2:**
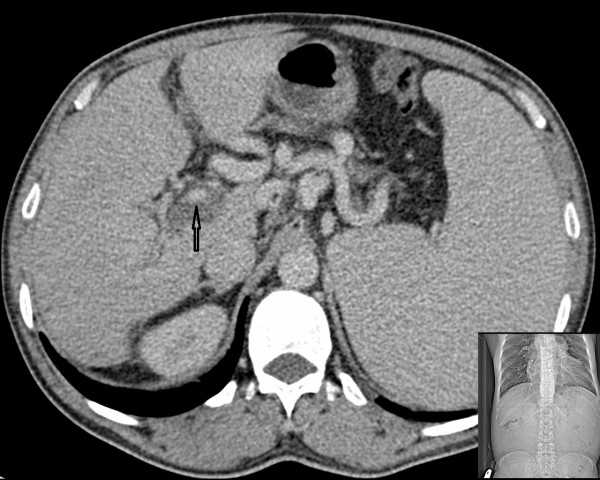
Contrast enhanced CT confirmed portal vein thrombosis formation.

This patient is then underwent open-heart aortic valve mechanical valve replacement under general anesthesia and cardiopulmonary bypass (CPB). Heparin resistance was noticed during CPB and resolved after administration of the fresh frozen plasma. He was extubated 24 hours after the procedure with stable hemodynamic status. Warfarin was administered orally for since 48 h after surgery to maintain the international normalized ratio (INR) at 1.5–2.0. In third postoperative day, patients developed pulmonary infection and increased body temperature with positive sputum culture of bacteria klebsiella pneumonia. Then after several days of intravenous antibiotics, his symptom relieved. On the fifth postoperative, edema in lower extremities was well as obvious ascites were noticed in this patients. Best side echocardiogram reveal normal mechanical valve function with LV ejection fraction 65%. Abdominal ultrasound revealed thrombosis in proximal portion of the portal vein with no blood flow signal as well as dilated distal branch of portal vein system (Figure 1). Contrast enhanced CT also confirmed portal vein thrombosis formation in this patient (Figure 2). Gastroscopy examination showed the obvious esophageal and gastric varices formation. Further coagulation screening text confirmed ATIII deficiency in this patient with only 30% of normal reference valve, this level still maintained 3 month after the surgery. Conservative treatment strategy was chosen for this patient including increased intensity of anti-coagulation (combining lower molecular heparin as well as oral warfarin) as well as endoscopic variceal ligation. Patient’s symptom gradually improved and repeated contrast enhanced CT scanning showed the formation of portal venous collateral circulation. This patients then underwent open abdominal thrombectomy 1 month after the cardiac procedure and recover well.

## Discussion

Portal vein thrombosis refers to the development of thrombosis within the extrahepatic portal venous system draining into the liver [3]. It is a rare but well-known complication after live surgery. Pathology such as myeloproliferative disorder, liver cirrhosis with portal hypertension, deficiency of natural anticoagulant proteins such as protein C or antithrombin III as well as hepatocellular carcinoma also could contribute to PVT. However, PVT after cardiac surgery is extremely rare and pose a truly a diagnostic and management challenge. It’s clinical signs are not specified and the symptoms of PVT may mimicking conventional complications after cardiac surgery such as lower limb edema as well as GI bleeding do to stress ulcer. Certainly fatal GI bleeding is one potential outcome after portal vein thrombosis along with other complications such as liver failure but fatal bleeding is probably not a forgone outcome. In this unique case, the possible causes for PVT after cardiac surgery may due to: Primary coagulant function abnormality with ATIII deficiency; the postoperative infection and bacteria; as well as the coagulation and hemostasis dysfunction caused by cardiopulmonary bypass (CPB). antithrombin III deficiency has been associated with the predisposition to thromboembolic disease [4].

There are many potential causes of lower extremity edema in patients with heart valve replacement, including portal vein thrombosis, elevated portal venous pressure from cirrhosis, congestive heart failure, constrictive pericarditis, nephrotic syndrome and peritoneal infections. As for patient with suspected PVT, favorable prognosis depends on rapid and accurate diagnosis which may facilitate by CT or ultrasound exam. Early intervention such as enhance the anticoagulant therapy, endoscopic variceal ligation, trans-jugular intrahepatic portosystemic stent shunt (TIPS) as well as surgical thrombectomy will also great improve the patient prognosis. As show in this patient with PVT, systemic anticoagulation with low molecular weight heparin and warfarin, Also, for this type of patient, endoscopic variceal ligation is also necessary as the initial treatment aimed to prevent further thrombosisand massive GI bleeding [1,3]. TIPS and portosystemic shunt surgery to decompress the portal venous system appears to be a good option and it is usually reserved for the treatment of variceal bleeding that had not responded to endoscopic treatment [5].

## Conclusions

PVT after cardiac surgery is a serious complication, which has only be reported in sporadic case. This case reveals that we should consider coagulation routine test related to antithrombin III deficiency before the operation. Thus, an appropriate diagnostic and treatment approach is desirable in an attempt to reduce morbidity and mortality.

## Consent

Written informed consent was obtained from the patient for publication of this case report and any accompanying images. A copy of the written consent is available for review by the Editor-in-Chief of this journal.

## Abbreviations

PVT: Portal vein thrombosis; ATIII: Antithrombin III; CPB: Cardiopulmonary bypass; EKG: Electrocardiogram; PT: Prothrombin time; APTT: Activated partial thromboplastin time; INR: International normalized ratio; LV: Left ventricle; CT: Computerized tomography; GI: Gastrointestinal; TIPS: Trans-jugular intrahepatic portosystemic stent shunt.

## Competing interests

The authors declare that they have no competing interests.

## Authors’ contributions

YQW wrote the draft of the manuscript and obtained the written consent. DZ and QLC participated in the manuscript writing and helped to the final writing of the paper and gave final approval of the manuscript. LD participated in the manuscript revision. All authors read and approved the final manuscript.
